# Increasing adiposity and the presence of cardiometabolic morbidity is associated with increased Covid-19-related mortality: results from the UK Biobank

**DOI:** 10.1186/s12902-021-00805-7

**Published:** 2021-07-03

**Authors:** Kiran H. K. Patel, Xinyang Li, Jennifer K. Quint, James S. Ware, Nicholas S. Peters, Fu Siong Ng

**Affiliations:** National Heart and Lung Institute, Imperial College London, 4th Floor, ICTEM Building, 72 Du Cane Road, London, W12 0NN UK

**Keywords:** Obesity, Adiposity, Covid-19, Mortality

## Abstract

**Background:**

Although obesity, defined by body mass index (BMI), has been associated with a higher risk of hospitalisation and more severe course of illness in Covid-19 positive patients amongst the British population, it is unclear if this translates into increased mortality. Furthermore, given that BMI is an insensitive indicator of adiposity, the effect of adipose volume on Covid-19 outcomes is also unknown.

**Methods:**

We used the UK Biobank repository, which contains clinical and anthropometric data and is linked to Public Health England Covid-19 healthcare records, to address our research question. We performed age- and sex- adjusted logistic regression and Chi-squared test to compute the odds for Covid-19-related mortality as a consequence of increasing BMI, and other more sensitive indices of adiposity such as waist:hip ratio (WHR) and percent body fat, as well as concomitant cardiometabolic illness.

**Results:**

13,502 participants were tested for Covid-19 (mean age 70 ± 8 years, 48.9% male). 1582 tested positive (mean age 68 ± 9 years, 52.8% male), of which 305 died (mean age 75 ± 6 years, 65.5% male). Increasing adiposity was associated with higher odds for Covid-19-related mortality. For every unit increase in BMI, WHR and body fat, the odds of death amongst Covid19-positive participants increased by 1.04 (95% CI 1.01–1.07), 10.71 (95% CI 1.57–73.06) and 1.03 (95% CI 1.01–1.05), respectively (all *p* < 0.05). Referenced to Covid-19 positive participants with a normal weight (BMI 18.5–25 kg/m^2^), Covid-19 positive participants with BMI > 35 kg/m^2^ had significantly higher odds of Covid-19-related death (OR 1.70, 95% CI 1.06–2.74, *p* < 0.05). Covid-19-positive participants with metabolic (diabetes, hypertension, dyslipidaemia) or cardiovascular morbidity (atrial fibrillation, angina) also had higher odds of death.

**Conclusions:**

Anthropometric indices that are more sensitive to adipose volume and its distribution than BMI, as well as concurrent cardiometabolic illness, are associated with higher odds of Covid-19-related mortality amongst the UK Biobank cohort that tested positive for the infection. These results suggest adipose volume may contribute to adverse Covid-19-related outcomes associated with obesity.

## Background

As of 28 August 2020, the coronavirus (Covid-19) pandemic resulted in over 24 million confirmed cases and 827,000 deaths worldwide [1], of which over 330,000 and 41,000 were in the UK [2], respectively. With accumulating global experience, risk factors for Covid-19 infection and predictors of outcomes are becoming increasingly recognised. Amongst these, a body mass index (BMI) within the overweight (25-30 kg/m^2^) or obese (> 30 kg/m^2^) categories and cardiometabolic morbidity have been associated with a more serious illness that necessitate hospitalisation and invasive ventilation [3–6]. Whether these risk factors also translate into higher mortality is less well characterised. Furthermore, although BMI is the most commonly used metric for categorising obesity, it is an insensitive measure of adiposity as it does not account for differences in body composition. Given that adipose tissue may serve as a reservoir for Covid-19 virus and thus a more severe course of illness [7], using alternative anthropometric indices that are more sensitive to adipose volume and its distribution to identify patients at risk of Covid-19-related death would be clinically helpful. However, as such data are not routinely collected before or during hospitalisation the association between Covid-19 outcomes and body composition remains unknown. Likewise, although metabolic syndrome has been identified as a risk factor influencing progression of the infection [8], the added risk of mortality conferred by its constituent parts, i.e. diabetes, hypertension and dyslipidaemia, has not been reported.

Uniquely, the UK Biobank is a longitudinal cohort study containing clinical and anthropometric data collected before the pandemic [9, 10] that is also linked to national Covid-19 laboratory test data through Public Health England [11]. This has the added advantage that any association between Covid-19 outcomes and anthropometric indices and morbidity, respectively, are unlikely to be the result of reverse causation. We therefore leveraged this data repository to investigate whether there was an association between Covid19-related mortality and anthropometric and cardiometabolic data that was recorded prior to the pandemic amongst the UK Biobank cohort.

## Methods

The UK Biobank [9] (https://www.ukbiobank.ac.uk/) comprises 502,505 participants who were recruited between 2006 and 2010 at one of twenty-two assessment centres and were aged 40–69 years at the time. The current mean age of the cohort is 68 ± 8 years (range 49–86 years, 44% male). All participants provided informed consent at the time of recruitment for their data to be used for research purposes. Sociodemographic, lifestyle, health and anthropometric data were collected at baseline and during recall, for which protocols are publicly available [12]. Definitions of comorbidity were based on the main and secondary International Classification of Disease (ICD)-10 summary diagnoses or operative procedures.The latest Covid-19 data release (03/08/2020) included results from 16/03/2020 to 26/07/2020 and was accessed under application number 48666.

Age- and sex- adjusted logistic regression analyses were used to identify whether anthropometric indices [BMI (kg/m^2^), waist:hip ratio (WHR) and body fat (%)] and c-reactive protein (CRP) were associated with Covid-19-related mortality. Chi-squared test was conducted to identify the association between concurrent morbidity and Covid-19-related mortality. T test or Fisher’s exact test were used to compare characteristics of males vs females testing positive for Covid-19, and of normal weight vs obese individuals. Statistical analyses were performed using Python version 3.6 (Python Software Foundation) and *P* < 0.05 was considered significant.

## Results

By 28 August 2020, 13,502 UK Biobank participants (mean age 70 ± 8 years, range 50–84 years, 48.9% male) had been tested for Covid-19 using nasopharyngeal swabs. Of these, 1582 tested positive (mean age 68 ± 9 years, 52.8% male) and 11,920 negative. Among those that tested positive, 305 died (mean age 75 ± 6 years, 65.5% male).

Males testing positive for Covid-19 had a higher mortality than females (OR 1.93, 95% CI 1.49–2.51, *p* < 0.0001), and tended to be older (69 ± 9 vs. 66 ± 9 years, *p* < 0.0001). Males that tested positive were also more likely to have hypertension, dyslipidaemia, arrhythmia, diabetes or angina than females. There was no difference in BMI between sexes. Comparisons between males and females testing positive are detailed in Table 1.

**Table 1:** Differences in mortality and co-morbidity amongst males and females testing positive for Covid-19

	Male	Female	Odds ratio (95% CI)	*P*
N	835	747	–	–
Died (n)	200	105	1.93 (1.49–2.51)	< 0.0001
Age (mean years ± SD)	69 ± 9	66 ± 9	–	< 0.0001
BMI (kg/m^2^, mean ± SD	28.9 ± 4.8	28.6 ± 6.1	–	0.32
Hypertension (n)	343	226	1.61 (1.31–1.98)	< 0.0001
Dyslipidaemia (n)	206	97	2.20 (1.68–2.86)	< 0.0001
Atrial fibrillation (n)	109	35	3.05 (2.06–4.58)	< 0.0001
Ventricular arrhythmia (n)	9	1	8.13 (1.36–89.41)	0.02
Diabetes (n)	150	74	1.99 (1.47–2.70)	< 0.0001
Stroke (n)	20	15	1.20 (0.63–2.29)	0.61
Angina (n)	123	54	2.21 (1.58–3.08)	< 0.0001

Male participants testing positive have a higher odds ratio for mortality than their female counterparts. Males testing positive were older and had a greater comorbidity, although there was no difference in BMI or history of stroke between sexes. Age and BMI were treated as continuous variables and compared between sexes using T test. All other variables were treated as categorical and compared using Fisher’s exact test

Referenced to normal BMI range (18.5 to < 25 kg/m^2^), participants with BMI ≥ 35 kg/m^2^ (obese II) had significantly greater odds for Covid-19-related mortality (OR 1.70, 95% CI 1.06–2.74, *p* < 0.05). The odds for Covid-19-related mortality amongst individuals with BMI < 18.5 kg/m^2^ (underweight), 25 to < 30 kg/m^2^, (overweight) or 30 to < 35 kg/m^2^ (obese I) did not reach statistical significance.

For every unit increase in BMI, WHR and percent body fat, the odds of death amongst the swab-positive participants increased by 1.04 (95% CI 1.01–1.07), 10.71 (95% CI 1.57–73.06) and 1.03 (95% CI 1.01–1.05), respectively (all *p* < 0.05, Table 2).

**Table 2:** Association of adiposity and cardiometabolic illness with Covid-19-related mortality

	Total tested	Positive swab	Positive swab (died)	Odds ratio [95% CI]	*P*
Underweight (%)	1.53	1.57	3.27	1.62 [0.28–9.42]	> 0.05^a^
Normal weight (%)	27.09	23.57	18.03	Reference	
Overweight (%)	40.95	41.94	39.67	0.98 [0.67–1.43]	> 0.05^a^
Obese I (%)	20.44	20.54	24.59	1.33 [0.87–2.01]	> 0.05^a^
Obese II (%)	9.98	12.36	14.42	1.70 [1.06–2.74]	< 0.05^a^
BMI (kg/m^2^)	28.26 ± 5.22	28.79 ± 5.39	29.66 ± 5.74	1.04 [1.01–1.07]	< 0.001^a^
Waist:hip ratio (WHR)	0.89 ± 0.09	0.90 ± 0.09	0.93 ± 0.09	10.71 [1.57–73.06]	< 0.05^a^
Body fat (%)	32.06 ± 8.66	32.13 ± 8.60	32.17 ± 8.86	1.03 [1.01–1.05]	< 0.05^a^
CRP	3.01 ± 4.79	3.21 ± 5.83	3.88 ± 6.69	1.02 [1.00–1.04]	> 0.05^a^
Hypertension (%)	36.21	35.45	54.75	2.68	< 0.001^b^
Dyslipidaemia (%)	19.03	19.03	29.18	2.05	< 0.001^b^
Atrial fibrillation (%)	8.44	8.48	15.08	2.36	< 0.001^b^
Ventricular arrhythmia (%)	0.84	0.55	0.98	2.22	> 0.05^b^
Diabetes (%)	12.05	14.12	22.95	2.16	< 0.001^b^
Stroke (%)	2.36	2.18	3.93	2.25	> 0.05^b^
Angina (%)	11.37	11.09	17.38	1.97	< 0.001^b^

Referenced to the normal weight category, participants with a BMI in the highest obesity category (obese II) demonstrate a greater odds ratio of Covid-19-related mortality. Similarly, concurrent cardiometabolic morbidity was also associated with higher mortality. Obesity was also classified as a categorical variable based on BMI (underweight, BMI < 18.5 kg/m^2^; normal weight, 18.5 to < 25 kg/m^2^; overweight, 25 to < 30 kg/m^2^; obese I, 30 to < 35 kg/m^2^; and obese II, ≥ 35 kg/m^2^). Anthropometric and clinical indices were treated as continuous variables. Logistic regression model adjusted for age and sex was used to calculate odds ratios for continuous variables ^(a)^. Chi-squared test was used for categorical variables ^(b)^

Swab-positive participants with pre-existing metabolic (diabetes, hypertension, dyslipidaemia) or cardiovascular morbidity (atrial fibrillation, angina) also had higher odds of death (Table 2). The association between CRP and mortality did not quite reach statistical significance (OR 1.02, 95% CI 1.00–1.04, *p* > 0.05).

Amongst those testing positive for Covid-19, individuals in the obese II category (BMI > 35 kg/m^2^) had greater odds ratios for hypertension, dyslipidaemia, atrial fibrillation, diabetes and angina than their normal weight (18.5 to  25 kg/m^2^) counterparts. There were no significant differences in age, or history of ventricular arrhythmia or stroke between these categories (Table 3).

**Table 3:** Differences in co-morbidity amongst normal weight (BMI 18.5 to < 25.0 kg/m^2^) and obese (BMI > 35 kg/m^2^) participants that tested positive for Covid-19

	Normal weight (BMI18- < 25 kg/m^2^)	Obese (BMI > 35 kg/m^2^)	Odds ratio (95% CI)	*P*
Number (n)	389	204	–	–
Age (mean years ± SD)	67 ± 9	68 ± 9	–	0.20
Hypertension (n)	84	119	5.08 (3.53–7.30)	< 0.0001
Dyslipidaemia (n)	42	64	3.78 (2.44–5.84)	< 0.0001
Atrial fibrillation (n)	24	29	2.52 (1.46–4.48)	0.0021
Ventricular arrhythmia (n)	5	1	0.38 (0.032–2.76)	0.67
Diabetes (n)	15	67	12.19 (6.71–22.19)	< 0.0001
Stroke (n)	6	5	1.60 (0.55–5.47)	0.52
Angina (n)	20	38	4.22 (2.40–7.38)	< 0.0001

Amongst those testing positive for Covid-19, participants with BMI > 35 kg/m^2^ (obese II) have greater odds ratios for hypertension, dyslipidaemia, atrial fibrillation, diabetes and angina than individuals with normal weight (BMI 18.5 to < 25.0 kg/m^2^). There were no differences between groups for stroke and ventricular arrhythmia. T test was used to test for differences in age between groups, and Fisher’s exact test for categorical variables

## Discussion

We show that increasing adiposity and pre-existing cardiometabolic morbidity are associated with higher Covid-19 related mortality amongst the UK Biobank population. Furthermore, participants with BMI > 35 kg/m^2^ had significantly higher odds of Covid-19 related mortality compared to those of a normal weight (BMI 18.5–25 kg/m^2^). As the participants of the UK Biobank represent a large sample of the British population that have undergone extensive phenotypic characterisation since 2006, these results can likely be extrapolated to the general population and are unlikely to be influenced by reverse causality. They also extend our current understanding of how these risk factors influence the course and severity of Covid-19 infection [4, 6, 13].

We demonstrate that central obesity, quantified by waist:hip ratio, is associated with Covid19-related mortality. We postulate that volume and distribution of adipose tissue may be a determinant of adverse outcomes observed in those with a high BMI. Covid-19 virus binds to the angiotensin converting enzyme 2 (ACE2) receptor for intracellular invasion [14, 15]. Given that ACE2 is highly expressed in adipose as well as pulmonary tissue, it is unsurprising that obesity has been linked to a more severe illness [16]. It is, therefore, plausible that adipose tissue exerts a deleterious effect in Covid-19 infection by acting as a functional reservoir of the virus [17]. Inflammation of visceral adipose depots, such as epicardial fat that is contiguous with the myocardium, may also contribute to adverse outcomes by stimulating an augmented local and/or systemic inflammatory reaction in individuals with higher volumes of adiposity [13, 18, 19]. We anticipate it may be possible to ascertain whether subcutaneous or visceral adipose volumes and their respective distributions are associated with adverse Covid-19 outcomes as this data becomes available in a sufficient number of UK Biobank participants.

Counterintuitively, being overweight or obese is associated with a lower risk of viral infection and pneumonia, and underweight with a higher risk, compared to normal weight individuals [20]. On the other hand, individuals with a normal weight by BMI, but who nonetheless have central obesity according to WHR, have a higher risk of bacterial, not viral, infections [20]. Reasons for the so-called “obesity paradox” are not well understood. However, this phenomenon does not appear to be borne out in Covid-19 infection; indeed, our results would support the contrary. Excess adiposity weakens the innate immune system and may impair the clearance of viral infections [21], resulting in a persistent and perhaps exaggerated immune response in the context of a novel zoonotic viral infection compared to other seasonal viruses.

Obesity is associated with reduced respiratory reserve volume, functional capacity, and pulmonary tissue compliance. Central obesity also compromises pulmonary function, particularly in the supine position due to diaphragmatic splinting. These mechanical features make obese individuals and those with excess central adiposity more vulnerable to respiratory infections. The association between obesity and severe Covid-19 infection has previously been shown to remain significant after adjusting for glycated haemoglobin (HbA1c) and lipid profile, suggesting that increased adiposity is a risk factor [6]. Given that the association between increasing adiposity and Covid-19 related hospitalisation was only partially attenuated, and not negated, by HbA1c and high-density lipoprotein (HDL) cholesterol [6] also supports the possibility that the adverse outcomes observed with concurrent obesity may be partly mediated by adiposity. This is consistent with other reports from other infections [22] and those that have linked unhealthy lifestyle choices and behaviours to a higher risk of Covid-19 [23]. We extend these findings by demonstrating that pre-existing metabolic perturbation (diabetes, hypertension and dyslipidaemia) and cardiovascular disease (atrial fibrillation and angina) confer higher risk of Covid-19-related mortality in the same cohort. Obese individuals also had greater morbidity burden than their normal weight counterparts in our study. Given that cardiometabolic illnesses and obesity are chronic low-grade inflammatory phenotypes, cytokine activation may be amplified in the context of Covid-19 infection and may explain the higher mortality [7, 24, 25]. Similar to excess adipose tissue, a higher expression of ACE-2 receptor and consequently a greater viral load [26, 27], impaired neutrophil chemotaxis and phagocytosis, increased furin and impaired T cell function are other mechanisms by which pre-existing metabolic perturbation may confer susceptibility to infection and adverse outcomes [28].

Despite the demonstrable utility of the UK Biobank for prognostication in Covid-19 infection, our analysis utilises data from middle-aged and older individuals. Therefore, the impact of adiposity and comorbidity on Covid-19 related outcomes in younger adults was not evaluated in our study. Although compelling, our analyses suggest an association, though not a direct causal link, between increasing adiposity and pre-existing morbidity and Covid-19-related mortality. A mendelian randomisation study may yield greater insight into the possible independent causal effects of adiposity in Covid-19 susceptibility and severity, given that obese participants had a greater comorbidity than their normal weight counterparts. We also cannot rule out changes in weight and adiposity from baseline during the course of follow-up of the UK Biobank.

## Conclusion

In summary, we show that increasing adiposity and cardiometabolic morbidity are associated with higher Covid-19-related mortality amongst the UK Biobank cohort that tested positive for the infection. Adipose volume may influence the severity of Covid-19-related outcomes.

## Data Availability

The dataset analysed during the current study are available in the UK Biobank repository, https://www.ukbiobank.ac.uk/. This can be accessed by formal application via the same link.

## Abbreviations

ACE2: Angiotensin converting enzyme 2
BMI: Body mass index
Covid-19: Coronavirus
CRP: C-reactive protein
HbA1c: Glycated haemoglobin
HDL: High density lipoprotein
OR: Odds ratio
WHR: Waist:hip ratio
